# Aplastic anemia secondary to nivolumab and ipilimumab in a patient with metastatic melanoma: a case report

**DOI:** 10.1186/s40164-018-0098-5

**Published:** 2018-03-20

**Authors:** D. E. Meyers, W. F. Hill, A. Suo, V. Jimenez-Zepeda, T. Cheng, N. A. Nixon

**Affiliations:** Department of Oncology, University of Calgary, 1331 29th St NW, Calgary, AB T2N 4N Canada

**Keywords:** Immune checkpoint blockade, Immunotherapy, PD-1, CTLA-4, Melanoma, Adverse events

## Abstract

**Background:**

Immune checkpoint blockade (ICB) is becoming an increasingly prevalent strategy in the clinical realm of cancer therapeutics. With more patients being administered ICB for a host of tumor types, the scope of adverse events associated with these drugs will likely grow. Here we report a case of aplastic anemia (AA) in a patient with metastatic melanoma secondary to dual ICB therapy. To our knowledge, this is only the second case of AA secondary to dual ICB in the literature, and the first to have a positive patient outcome.

**Case presentation:**

A 51-year old male with metastatic melanoma was started on dual immune checkpoint blockade, in the form ipilimumab (3 mg/kg) and nivolumab (1 mg/kg). Two weeks following the second cycle, he presented to the emergency department with profound polypipsia, polyuria and fatigue. The patient was diagnosed with diabetic ketoacidosis secondary to immune therapy induced type-1 diabetes and was admitted to the ICU. While in hospital the patient developed a symptomatic anemia and neutropenia. A bone marrow biopsy revealed a markedly hypocellular marrow with trinlineage hypoplasia with no evidence of myelodysplasia, neoplasm or excess blasts. Flow cytometry revealed an inverted CD4^+^:CD8^+^ ratio and an absence of hematogones. Taken together the presumed etiology was AA secondary to immunotherapy. The patient was subsequently started in IV methylprednisone 70 mg/day for 8 days, followed by a prednisone taper. This intervention rectified the bicytopenia and to date the patient has shown stable blood counts.

**Conclusion:**

With the use of ICBs becoming increasingly prevalent in the clinical arena, the number of patients presenting with immune-related adverse events will likely increase. The current case illustrates the need to be vigilant when managing cancer patients receiving ICB. The resolution of this patient’s AA with corticosteroids highlights the value of early detection and appropriate treatment of these rare immune-mediated adverse events.

## Background

The last decade of concerted research efforts have led to a paradigm shift in the way we think about malignancy, with ‘avoiding immune destruction’ now recognized as a hallmark of cancer [1]. As such, immunotherapy has risen to the forefront as a therapeutic strategy of interest for a number of cancer types. Targeting immune suppressive checkpoint proteins, in particular cytotoxic T-lymphocyte antigen 4 (CTLA-4) and programmed death-1 (PD-1/PD-L1), has seen substantial clinical success in recent years. Immune checkpoint blockade (ICB) is recognized to cause frequent immune-related adverse events (irAEs) [2], which more commonly occur when combining agents. However, cytopenias secondary to ICB do not frequently occur. Here we report a case of aplastic anemia (AA) secondary to combined ICB in a patient with metastatic melanoma. To our knowledge, this is only the third reported case of AA secondary to ICB in the literature (Table 1). Our case is only the second in the context of dual ICB, and the first with a positive patient outcome.

**Table 1:** Summary of the three available cases of aplastic anemia in the setting of immune checkpoint blockade

	Case 1 (present case)^a^	Case 2 [8]	Case 3 [9]
Gender/age	M/51	F/48	F/57
Oncologic Dx	Metastatic melanoma	Metastatic melanoma	Metastatic GBM
Baseline CBC			
*Hb* (g/L)	160	N/A	~ 130
*Plt* (× 10^9^/L)	250	N/A	268
*Neut* (× 10^9^/L)	2.9	N/A	~ 2.5
ICB agent/dose	Nivolumab (1 mg/kg) + ipilimumab (3 mg/kg) × 2 cycles	Nivolumab (1 mg/kg) + ipilimumab (3 mg/kg) × 4 cycles, N (3 mg/kg) × 5 cycles	Nivolumab (3 mg/kg) × 2 cycles
Presentation of AA	15-days post 2nd cycles of nivolumab + ipilimumab	3-days post 5th cycle of nivolumab	~ 14-days post 2nd cycle of nivolumab
CBC @ AA presentation			
*Hb* (g/L)	77	115	68
*Plt* (× 10^9^/L)	346	< 5	5
*Neut* (× 10^9^/L)	0.06	< 0.1	0.00
BM biopsy	< 10% cellularity with trilineage hypoplasia without excess blasts, myelodysplasia, myeloid/lymphoid precursors or a B cell neoplasm. Lymphocyte fraction 84% T cells with an inverted CD4^+^:CD8^+^ ratio (1:2)	~ 10% cellularity, scattered lymphoid and erythroid cells without signs of dysplasia. Absent granulopoiesis and megakaryocytes missing. Majority of lymphoid cells were CD8^+^ T-lymphocytes	Markedly hypocellular marrow with virtual absence of hematopoietic elements. ~ 50% of cells were lymphocutes; majority T-cells. One analyzable metaphase; chromosomally normal
Treatment	Methylprednisone 1 mg/kg q 12 h × 7 days, 1 mg/kg q 24 h × 7 days, packed red blood cells	Prednisone 1 mg/kg/24 h, G-CSF, infection prophylaxis, tranexamic acid, platelet transfusions	Dexamethasone 2 mg PO q 12 h, G-CSF, eltrombopag 50 mg PO q 24 h → 100 mg PO q 24 h, platelet transfusions, packed red blood cells, infection prophylaxis
AA response/outcome	Rapid recovery in neutropenia, gradual recovery in hemoglobin	No response to treatment	No response to treatment
Patient outcome	No current active disease, patient being monitored	Patient mortality at day 11 of hospitalization from intracerebral hemorrhage	Patient mortality 73 days after cycle 2 of Nivolumab

*I* ipilimumab, *N* nivolumab

^a^Patient received one 200 mg dose of lomustine ~ 7 weeks prior

## Case presentation

A 51-year-old Caucasian man being treated with ipilimumab (3 mg/kg) and nivolumab (1 mg/kg) for metastatic melanoma presented to the emergency department with profound polydipsia, polyuria and increasing fatigue. Clinical workup led to the diagnosis of diabetic ketoacidosis (DKA), presumed secondary to immune-therapy induced type-1 diabetes. Further, the patient was also noted to have a normocytic anemia (88 g/L, MCV 82 fL), and neutropenia (0.06 × 10^9^/L). The platelets were measured within normal limits at 346 × 10^9^/L, and reticulocytes were 2%. The patient was admitted to the intensive care unit (ICU) for DKA management—including intravenous fluid resuscitation and insulin therapy—and evaluation of the bicytopenia.

The patient had originally been referred to our cancer centre 8 years previous with a diagnosis of BRAF-wildtype stage III malignant melanoma of the left thigh. Initial therapy consisted of surgical resection, partial local lymph node dissection and interferon for 11 months. Two years later the disease recurred, involving contralateral groin lymph nodes. Bilateral lymph node dissection was performed and adjuvant radiotherapy was administered. One year later, a metastatic lesion to the right orbit was discovered and subsequently resected, followed by adjuvant radiotherapy.

The patient remained well until this year, when a suspected subcutaneous metastasis at the level of the C7 spinous process was discovered and confirmed on magnetic resonance imaging (MRI). MRI also showed a left parieto-occipital lobe lesion measuring 2.1 cm. The recommended therapeutic strategy was radiosurgery to treat the intracranial metastasis, as well as systemic therapy in the form of dual ICB with ipilimumab and nivolumab. The first cycle was administered without incident (day 0) and on day 19, cycle two was administered. On day 35, the patient presented to the emergency department with symptoms of DKA. A formal assessment of disease response to ICB was not undertaken, as the patient hadn’t completed the induction phase. However, a brain MRI undertaken at admission suggested stable disease based on RECIST [3] criteria.

Although the DKA was controlled in the ICU, the cytopenias persisted. As such, the patient was transfused with 1 unit of packed red blood cells (PRBC) on day 41 for symptomatic relief of their anemia. Blood counts on day 42 demonstrated Hb 98 g/L, neutrophils 0.6 × 10^9^/L and platelets 518 × 10^6^/L. The mild thrombocytosis was thought to be reactive, since ferritin was also elevated (1228 pmol/L) at this time. Parvovirus serology was performed and was negative, whilst a positive direct antiglobulin test (DAT) was noted. However, DAT have reported to be positive in patients receiving monoclonal bodies like ipilimumab and nivolumab [4]. Further, although there was a nonspecific rise in LDH, there were no noted abnormalities in haptoglobin or bilirubin. As such, the DAT was not felt to be indicative of an autoimmune hemolytic anemia. Further, the patient had no prior history of autoimmune diseases, cytopenias or congenital bone marrow failure. Family history was non-contributory.

**Fig. 1:**
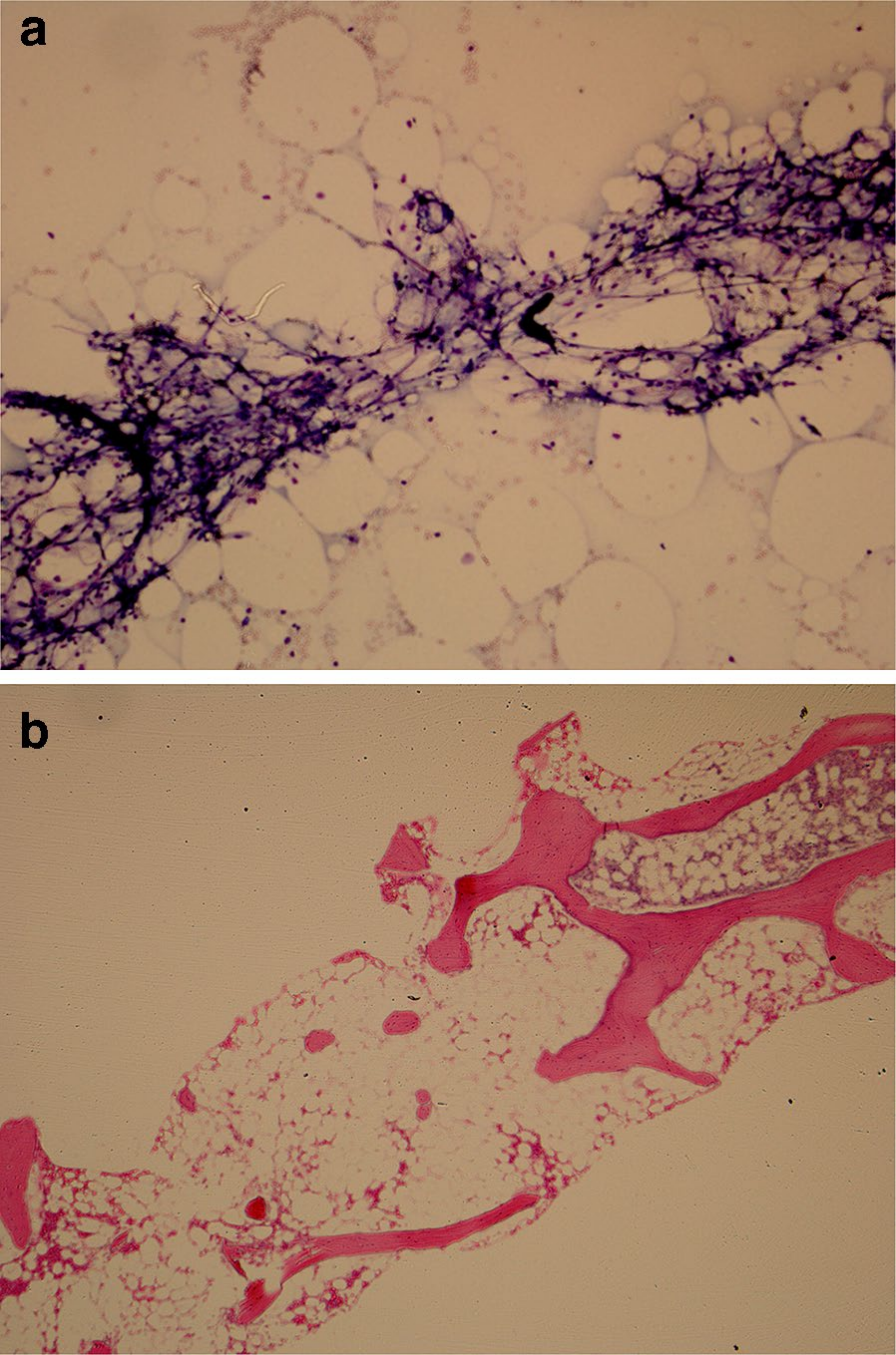
Patient bone marrow **a** aspirate and **b** biopsy. **a** Aspirate demonstrates spicules composed of stromal components, but lacking trilineage marrow elements. **b** Biopsy reveals a hypocellular marrow with global trilineage hypoplasia

On day 48 a bone marrow biopsy was performed, which showed a markedly hypocellular marrow (< 10%) with trilineage hypoplasia (Fig. 1). There was no morphological evidence of excess blasts or myelodysplasia, and no immunophenotypic evidence of elevated myeloid/lymphoid precursors or a B cell neoplasm. The lymphocyte fraction was composed of 84% T cells with an inverted CD4^+^:CD8^+^ ratio (1:2). Furthermore, flow cytometry showed an absence of hematogones, consistent with AA. Taken together, the presumed etiology for the clinical presentation was AA secondary to immunotherapy.

**Fig. 2:**
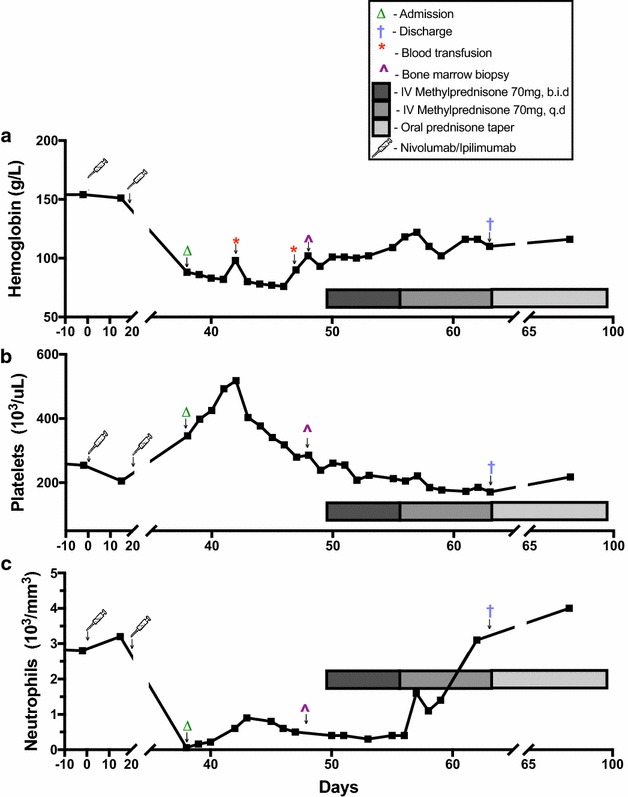
Trends in patient **a** hemoglobin, **b** neutrophils and **c** platelets over time

From day 49 to day 55 the patient was administered intravenous methylprednisone 1 mg/kg q 12 h, which was reduced to 1 mg/kg q 24 h from day 55 to day 63. The patient responded well to the steroid therapy, with a marked recovery in both hemoglobin and absolute neutrophil count (Fig. 2), providing support for the presumed diagnosis. The patient was discharged with the plan to continue the prednisone taper over the following 7 weeks with bi-weekly blood panels. Due to the development of two presumed grade 3/4 drug-mediated auto-immune complications, the patient was not restarted on ipilimumab or nivolumab. Follow-up completed to date has demonstrated continued stability in hemoglobin, neutrophils and platelets. As such, a repeat bone marrow biopsy has not been undertaken. The patient has since received stereotactic radiation to the intracranial metastasis and surgical resection of the subcutaneous lesion. At this time, there are no other sites of metastatic disease, and the patient is under active surveillance.

## Discussion

Options for the therapeutic management of metastatic melanoma have drastically changed over the past decade since it was demonstrated that inhibiting CTLA-4 with the monoclonal antibody ipilimumab could significantly enhance survival in these patients [5]. More recently, a landmark phase III clinical trial demonstrated that the addition of nivolumab, a monoclonal antibody to PD-1, led to a 3-year overall survival rate of 58%, as compared to 34% with ipilimumab monotherapy [6].

As previously stated, ICB—especially when used in combination—have the potential to cause a wide array of AEs, but hematologic AEs do not commonly occur. Specifically, in a pooled dataset of 448 melanoma patients treated with both nivolumab and ipilimumab [7], isolated grade 3 or 4 anemia, neutropenia and thrombocytopenia were reported in 2.8, 0.7 and 1.3% of patients, respectively. Furthermore, AA in the setting of ICB is an extremely rare occurrence, and has only been reported in the literature twice before [8, 9], with one case occurring secondary to single-agent nivolumab [9].

While CTLA-4 inhibitors affect the control of T cell priming centrally, and PD-1 inhibitors manipulate T cell activation peripherally (i.e. in the tumor microenvironment), both ultimately act to increase T cell mediated anti-tumor immunity. Through these mechanisms, though, T cell activation can inappropriately occur against self-antigens leading to auto-immune disease. Theoretically, T-cell activation can also occur against early hematopoietic progenitors, which could lead to an immune-mediated AA; as was seen in our patient. There are a number of known aberrancies that can contribute to the T cell-mediated destruction of hematopoietic progenitors, including oligoclonal expanded populations of autologous T cells, Th1 polarization leading to production of inhibitory cytokines, and Th17 immune responses [10]. More generally, the pathophysiology of AA can be attributed to aberrantly expressed T-cell receptor molecules, including the down-regulation of immune checkpoints CTLA-4 and PD-1 [11]. Therefore, in our patient case, the inappropriate modulation of T-cell activity secondary to treatment with nivolumab and ipilimumab could certainly have lead to the observed AA.

As our patient was noted to have a bicytopenia, including anemia and neutropenia, a bone marrow biopsy was indicated. Combined therapy with both nivolumab and ipilimumab could lead to inappropriate priming of CD8^+^ effector T cells against self antigen, specifically those of hemaptopoietic precursors. Both the findings of an elevated T:B lymphocyte ratio in the presence of a globally hypocellular marrow, as well as an inverse CD4^+^:CD8^+^ T cell ratio would support that rationale. Further supportive evidence of the presumed etiology came from the recovery of the patients’ blood counts in response to treatment with steroids. Curiously, there was never any clinical evidence of thrombocytopenia—as would likely be expected with an AA. As our patient was originally admitted to hospital with DKA secondary to immunotherapy-mediated diabetes, it is plausible that the induced pancreatic inflammation led to the elevation of both ferritin and platelets as acute phase reactants. A link between diabetes and elevated serum ferritin has previously been established [12], so although speculative this mechanism is certainly possible. Further, it is commonly understood that the average lifespan of a platelet is ~ 8–10 days. As indicated in Fig. 2c, the patient has an initial rise in platelet levels subsequent to receipt of nivolumab and ipilimumab, which would correspond to their admission to hospital with DKA. Once the ICBs were discontinued, the platelet levels drop over ~ 10 days to a nadir that was lower than their baseline. As such, this course would correspond with an increase in platelets as an acute phase reactant, and then a drop as the bone marrow began to fail. It is interesting to note that although the bone marrow cellularity was only ~ 10% there were normal megakaryocytes in the limited areas of hematopoiesis.

Data collected from pre-clinical and clinical studies over the past decade have allowed for the transition of ICBs, like ipilimumab and nivolumab, into the clinical arena. With US Food and Drug Administration/Health Canada approval currently in place for ICBs across a number of tumor types (i.e. melanoma, non-small cell lung cancer, renal cell carcinoma and urothelial cancer), and with more expected in the coming years, it is prudent for both patients and health care practitioners to understand the scope of possible AEs that may occur with their use. Fortunately, early recognition, diagnosis and initial treatment with systemic corticosteroids lead to resolution of most irAEs within 6–12 weeks [13]. Severe grade 3 or 4 AEs, however, typically require hospitalization, involvement of organ-specific specialists and possibly other immunosuppressive drugs such as tumor necrosis factor-alpha antagonists and/or azathioprine [13]. In the case of our patient, admission to the ICU for DKA management, consultation with hematology to workup the bicytopenia and prompt initiation of IV methylprednisone led to a favorable outcome. As the only other reported case of AA in the setting of dual ICI therapy led to a patient fatality [8], this highlights the potential severity of such AEs. Although the standard of care for treatment of AA typically includes immunosuppressive therapies like anti-thymocyte globulin and cyclosporine, prednisone was initiated in hopes of treating the underlying etiology.

To our knowledge, this is the first reported case of successfully treated AA secondary to dual ICB. The current case illustrates the need to be vigilant when dealing with cancer patients receiving ICB. Although most patients receiving these agents have some expected AE profile, unexpected and serious AEs can occur, and require early recognition and urgent management. Proper evaluation, either in the primary care setting, the emergency department, or by a specialist, and subsequent administration of corticosteroids is imperative. With the use of ICBs and other immununotherapies becoming increasingly common for the treatment of numerous malignancies, the number of patients presenting with irAEs will increase. Interestingly, two recent studies published by Du et al. [14, 15]. demonstrate that the anti-tumor effects of CTLA-4 blockade occur through independent mechanisms of those that contribute to irAEs, and that directly blocking the CTLA-4 axis with monoclonal antibodies like ipilimumab may not be necessary to mediate anti-tumor immunity at all. Ultimately, time will dictate whether we will be able to develop safer and more efficacious ICB based on this new paradigm.

## Conclusions

- Immune checkpoint inhibitors such as ipilimumab and nivolumab are becoming more frequently used in the treatment of a variety of malignancies.
- Most patients treated with ICIs will have mild AEs, but grade 3/4 can occur in a subset of patients.
- Early recognition of possible irAEs by patients and their health care providers and subsequent treatment with corticosteroids is important.
- Rare and generally unreported irAEs (like AA) can occur and specialists should be consulted to assist with diagnosis and management.

## Abbreviations

AA: aplastic anemia
irAEs: immune-related adverse events
CTLA-4: cytotoxic T-lymphocyte antigen 4
DKA: diabetic ketoacidosis
ICB: immune checkpoint blockade
PD-1: programmed death-1
